# Recent Advances in Biofilm Control Technologies for the Food Industry

**DOI:** 10.3390/antibiotics14030254

**Published:** 2025-03-01

**Authors:** Jirapat Dawan, Song Zhang, Juhee Ahn

**Affiliations:** 1 Department of Biomedical Science, Kangwon National University, Chuncheon 24341, Republic of Korea; jirapat@kangwon.ac.kr (J.D.); ed5988449@kangwon.ac.kr (S.Z.); 2 Department of Food Science and Nutrition, Zhejiang University, Hangzhou 310058, China; 3 Future Food Laboratory, Innovation Center of Yangtze River Delta, Zhejiang University, Jiaxing 314100, China

**Keywords:** anti-biofilm, antibiotic, antimicrobial, bacteriophage, probiotic, thermal processing, surface modification

## Abstract

Biofilms remain a major challenge in the food industry due to the increased resistance of foodborne pathogens to antimicrobial agents and food processing stresses, leading to food contamination and significant health risks. Their resistance to preservation techniques, antimicrobial treatments, and processing conditions increases concerns regarding food safety. This review discusses recent developments in physical, chemical, and surface modification strategies to control and remove biofilms in food processing environments. Physical methods, such as thermal treatments, electric fields, and ultrasonic systems, have demonstrated their efficacy in disrupting biofilm structure and improving disinfection processes. Chemical treatments, including the use of sanitizers, disinfectants, acidulants, and enzymes, provide targeted approaches to degrade biofilm matrices and inhibit bacterial adhesion. Furthermore, surface modifications of food contact materials provide innovative solutions for preventing biofilm formation and enhancing food safety. These cutting-edge strategies not only improve food safety but also reduce contamination risk in food processing facilities. The review highlights the mechanisms, efficacy, and applicability of these techniques, emphasizing their potential to mitigate biofilm-associated risks and ensure food quality and safety.

## 1. Introduction

Food safety has become a major public health issue due to food spoilage and foodborne illnesses caused by pathogens [1,2]. Food contamination can arise throughout any stage of production and handling, originating from human contact, animal sources, or environmental factors [3]. Foodborne illnesses lead to a variety of health problems, including neurological, immunological, and gastrointestinal disorders [4]. Food surfaces and processing equipment are commonly contaminated, often leading to the spread of foodborne pathogens. Several methods have been developed to preserve food and extend shelf life such as chilling, freezing, vacuum packing, salting, and the use of chemical preservatives [5,6]. Although these methods effectively reduce the risk of contamination, certain bacteria can still survive under these conditions. Bacteria exposed to preservation-related stresses may persist in a dormant state or as metabolically inactive cells such as viable but non-culturable (VBNC) cells or persister cells [7]. Additionally, bacteria can form biofilms as a survival strategy in response to harsh environments [8]. A biofilm is a structured community of microorganisms, including bacteria, fungi, and algae, that adhere to a surface and are embedded within a self-produced extracellular polymeric matrix [9]. This matrix provides structural stability, supports nutrient exchange, and ensures protection against environmental stresses such as antimicrobial agents and immune system responses [9].

Biofilms can form on a wide range of surfaces commonly found in food processing and storage environments, including stainless steel, plastic, rubber, and other food contact materials [10]. The presence of biofilms in food systems raises significant concerns for food safety and quality, as they can harbor pathogenic and spoilage microorganisms [11]. Biofilm formation involves several stages: initial attachment of bacteria to food surfaces, followed by proliferation, production of extracellular polymeric substances (EPSs), maturation of the biofilm, and eventual release of bacterial cells [9]. The EPS matrix, composed of polysaccharides, proteins, lipids, and nucleic acids, protects bacteria from external threats and supports their growth [12]. This matrix contributes to the persistence of biofilms in food processing environments [13].

In food production facilities, biofilms are difficult to eliminate despite regular cleaning and sanitization [13]. Foodborne bacteria can adhere to various surfaces, forming biofilms that allow them to survive for long periods, depending on the bacterial strain and environmental conditions [14]. One of the main challenges in managing biofilms is their resistance to antimicrobials and disinfectants. Bacteria within biofilms are more resilient, making them harder to remove with conventional cleaning methods [12]. Biofilms provide several benefits to bacteria, including protection from chemicals, resistance to mechanical stress caused by liquid flow in pipelines, and protection against desiccation [13]. Biofilms are a concern across various food industries, including seafood, poultry, dairy, brewing, and meat processing [4]. In addition, the rise of antibiotic-resistant bacteria has led to more biofilm-related contamination and infections [4]. New strategies for controlling biofilms in food production environments need to be developed to ensure food safety. This review discusses recent advancements in biofilm control methods, focusing on their mechanisms, effectiveness, and practical applications in mitigating biofilm-related risks in food processing.

## 2. Physical Strategies for Controlling Bacterial Biofilms in the Food Industry

In recent years, extensive research has focused on the development and application of physical strategies for controlling bacterial biofilms, which are resistant and difficult to remove from surfaces in diverse industries, particularly in food processing environments [15,16]. Biofilms cause significant risks by protecting pathogens, reducing the effectiveness of conventional cleaning and disinfection methods. Consequently, physical methods have received increasing attention due to their ability to disrupt biofilm structures and prevent their formation. These approaches, including thermal processing, electric fields, ultrasound, and surface modifications, provide distinct advantages and can be optimized for specific applications. Each strategy plays an essential role in improving biofilm control, enhancing sanitation practices, and ensuring the safety and quality of food products (Table 1).

### 2.1. Thermal Processing for Biofilm Removal in Food Processing Environments

**Figure 1:**
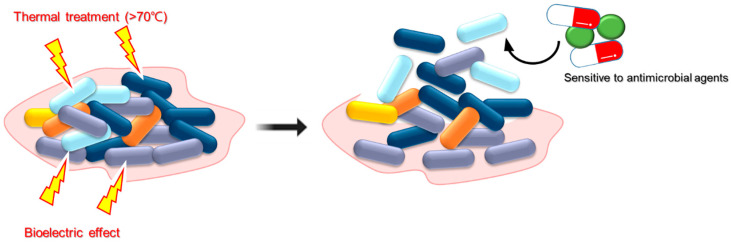
Thermal treatment and bioelectric effects for biofilm removal.

Thermal treatment is widely recognized as an effective method for controlling bacterial biofilms in food processing environments. The application of heat disrupts microbial cells by denaturing proteins, damaging nucleic acids, and altering membrane integrity, ultimately leading to microbial inactivation [11]. Thermal treatment disrupts the structural integrity of biofilms, making them more vulnerable to subsequent cleaning and disinfection processes by exposing equipment surfaces to high temperatures (typically above 70 °C) (Figure 1) [29]. For instance, hot water (71 °C, 30 s) treatments have been demonstrated to effectively eliminate biofilm cells up to 7 log CFU/cm^2^ [18]. Similarly, the inactivation of biofilms on diverse food contact surfaces such as high-density polyethylene and polypropylene can be accomplished extremely well using superheated steam (SHS) treatment (150 °C) [17].This thermal intervention not only removes existing biofilms but also inhibits their reformation, ensuring the safety and quality of food products [29]. Additionally, thermal processing is a flexible method that works effectively with a wide range of equipment types and materials commonly used in food processing plants [11]. Thermal processing, known for its effectiveness and practicality, remains essential in maintaining high hygiene standards and reducing the risks associated with biofilm contamination in the food industry. However, despite its effectiveness, heat treatment involves certain disadvantages that warrant consideration. One notable limitation is its potential to cause damage to sensitive components [30]. Excessive heat exposure can cause the degradation of certain materials, compromising their functionality and shelf life. Moreover, the energy-intensive nature of thermal processing also leads to increased operating costs, creating economic challenges for food processing facilities, especially those operating on a large scale [7]. Balancing the benefits of biofilm control with the associated drawbacks of heat treatment requires careful consideration of equipment compatibility, operational parameters, and cost-effectiveness. Nevertheless, advancements in heat treatment technologies and strategies provide promising opportunities to mitigate its limitations and enhance its effectiveness [11]. Innovations such as precise temperature control, targeted heating methods, and integrated monitoring systems enable more efficient and sustainable biofilm control practices in the food industry [30,31]. By utilizing these advancements and adopting a tailored approach to meet specific operational needs, food processing operations can optimize the benefits of thermal processing while reducing its drawbacks, ultimately ensuring the sustained safety and integrity of their products.

### 2.2. Bioelectric Effects for Enhanced Biofilm Control in Food Processing Applications

Electric fields may have an impact on the organization of biological membranes, cell behavior, metabolic processes, and the cellular responses of both prokaryotic and eukaryotic cells [32]. Bacterial adherence to the surface may be mediated by repulsive electrostatic forces. Studies have showed that a low-frequency, low-voltage alternating current (AC) can promote bacterial cells to exhibit increased activity, thus strengthening surficial attachment instead of biofilm formation [19]. The increased repulsive forces by electric fields facilitate the detachment of bacterial biofilms from surfaces. This phenomenon is called the electricidal effect [20]. Furthermore, this electric field can be used in combination with biocides or antimicrobials and pose a synergistic effect, a phenomenon called the bioelectric effect (Figure 1) [20]. Potential mechanisms of bioelectric effects on biofilm control may include increased membrane permeability, electrophoretic enhancement of antimicrobial agent transport, electrolytic production of oxygen, and electrochemical production of oxidants [33]. Low-intensity direct current (DC) has demonstrated the ability to detach bacteria from stainless steel and indium tin oxide surfaces, and this ability may be related to electrical effects [20]. Low-amperage current or 4 h/day of intermittent current can reduce *S. aureus*, *S*. *epidermidis*, and *P. aeruginosa* biofilms on a variety of surface materials [21]. This implies that low doses of DC can reduce biofilm formation [34]. In addition to electric field intensity, exposure time is also an important factor that affects anti-biofilm activity. Prolonged exposure to a low-intensity electric field significantly reduced the biofilm formation and bacterial cell density of both Gram-positive and Gram-negative bacteria. This finding indicates that sustained application of an electric field can effectively inhibit biofilm formation or promote its degradation [35]. However, the combined use of electric current and antimicrobials produced a bioelectric effect that was more effective in controlling the biofilms. The biofilm numbers of *E. coli* and *Streptococcus gordonii* were more decreased in the combination treatment between gentamicin and electric current than single treatment [7]. A correlation has been demonstrated between the therapeutic effect of electrical energy and bioelectric effects on biofilm prevention. The effectiveness of biofilm prevention treatment primarily depends on the energy of the electrical signal [7].

### 2.3. Applications of Ultrasound for Biofilm Disruption in Food Industry

**Figure 2:**
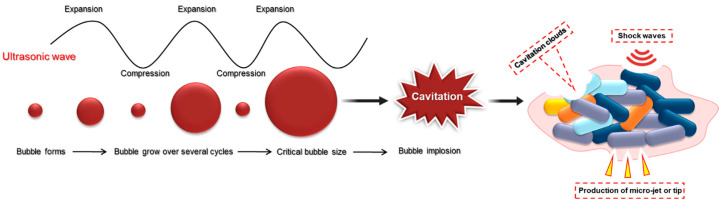
Ultrasonic systems for the removal of biofilms.

Ultrasound is sound with a frequency level above the human hearing threshold of 20 kHz [36]. Ultrasound can generate cavitation bubbles, which are known to contribute to erosion and cause damage to the target. The effect of cavitation bubbles can break up and detach bacterial biofilm from a surface [37]. There are four distinct mechanisms through which ultrasound exhibits bactericidal activity against bacteria and biofilms: (i) acoustic cavitation, (ii) enhanced antimicrobial activity, (iii) mechanical oscillation of a tip, and (iv) heat generation [36,38]. Figure 2 illustrates the process of cavitation induced by ultrasonic waves, which involves alternating phases of compression and expansion in a liquid medium [39]. During the expansion phase, the pressure decreases, leading to the formation of microscopic gas bubbles. These bubbles grow through successive cycles of pressure variation until they reach a critical size, at which point they become unstable and collapse [36]. The implosion of these bubbles releases substantial amounts of energy, generating localized high temperatures and pressures. The resulting cavitation clouds, formed by numerous collapsing bubbles, produce micro-jets and shock waves that can cause physical disruption of surfaces, enhance mixing, and facilitate microbial inactivation or biofilm removal [36]. This phenomenon has broad applications in fields such as cleaning, emulsification, and biofilm disruption [40]. Low-frequency ultrasound (typically <500 kHz) is significantly more effective than high-frequency ultrasound (>500 kHz) in reducing bacterial viability within biofilms, particularly when considering biofilm thickness and surface characteristics such as such as stainless steel, glass, and pipes [38,39]. Low-frequency ultrasound produces more intense cavitation and larger cavitation bubbles because the energy required to initiate cavitation in the liquid decreases as the frequency is reduced [38]. Thus, biofilms may be more successfully removed by strong cavitation implosion at low-frequency ultrasound. Similarly, ultrasonic waves in combination with various organic acids can effectively detach surficial bacterial cells and exhibit potent antibiofilm activity [23]. In addition, since ultrasonic waves are mechanical waves, ultrasonic processing also exhibits significant agitation, shear stress, and turbulence [36]. The shear force generated by ultrasound can also be used to remove biofilm from surfaces [40]. Besides the bactericidal effect on biofilms by ultrasound, it also significantly alters biofilm adhesion. The detachment of biofilm from a contact surface is one of the main purposes for adopting ultrasound in the food industry. For example, flat and curved ultrasonic transducers have been developed for removing biofilms in milk from opened and closed surfaces, respectively [38]. The detachment of bacterial cells is related to the cavitation effect produced by low-frequency ultrasound [38]. In addition, high-intensity ultrasound can lyse cell membranes, killing cells and partially detaching them from the surface [38]. Consequently, in the food industry, low-frequency and high-intensity ultrasound might be a useful tool for controlling foodborne bacteria biofilms [41]. Several challenges must be considered when using ultrasonic waves for biofilm control. One key limitation is the lack of directivity, meaning ultrasonic waves may unintentionally affect surrounding materials such as food and equipment. This can lead to protein denaturation, material degradation, or structural damage. Additionally, ultrasonic waves alone are not always effective in completely eradicating bacterial cells. Surviving bacteria may persist and potentially form new biofilms, resulting in further contamination.

### 2.4. Promising Surface Modification Strategies for Biofilm Control in Food Equipment

**Figure 3:**
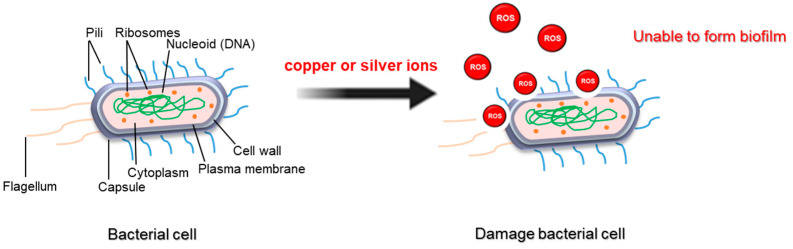
Reactive oxygen species produced by surface modification agents inhibit biofilm formation in bacteria.

Surface modification of contact materials is an effective strategy for biofilm control in the food industry, providing innovative solutions to reduce bacterial colonization and enhance hygiene standards [42]. Possible surfaces that are less prone to biofilm formation can be created by altering the surface properties of materials commonly used in food processing equipment, such as stainless steel or plastics, through techniques like coating deposition, surface texturing, or chemical treatments. These improvements include decreasing surface roughness to reduce attachment points for bacteria, introducing antimicrobial agents to inhibit bacterial growth, or adding hydrophobic coatings to repel water and organic matter [42]. For instance, the utilization of food-safe oil-based slippery coatings (FOSCs) improved the surface roughness, thus enabling the significantly decreased adhesion of bacteria and biofilm contamination [26]. This customized surface modification not only prevents initial bacterial adhesion but also facilitates easier cleaning and sanitization, improving overall equipment hygiene and minimizing the risk of food contamination. Compounds containing copper or silver are permitted for use in food products according to the Biocidal Products Regulation (BPR). In detail, excellent antibiofilm efficacy has been observed when the surface of the material was coated with copper or silver, ultimately allowing significant elimination against biofilm cells [27,28]. Copper ions are known to interact with bacterial cell membranes, causing DNA damage by forming reactive oxygen species (ROS) and interfering with the ability to form biofilms (Figure 3) [43]. However, the use of copper material is limited due to the high cost, potential loss of copper ions, and corrosion [43]. Sliver ions can also induce the formation ROS, which can generate oxidative stress in bacteria and destroy the permeability of bacterial cell membranes [42]. However, surface modification is often too complicated, including a variety of organic compounds, solvents, and chemical reactions. Although antifouling capabilities have been shown to be promising in vitro, their application in food processing systems should be extensively examined to ensure food safety [42].

## 3. Chemical Treatments in Food Processing and Biofilm Control

**Figure 4:**
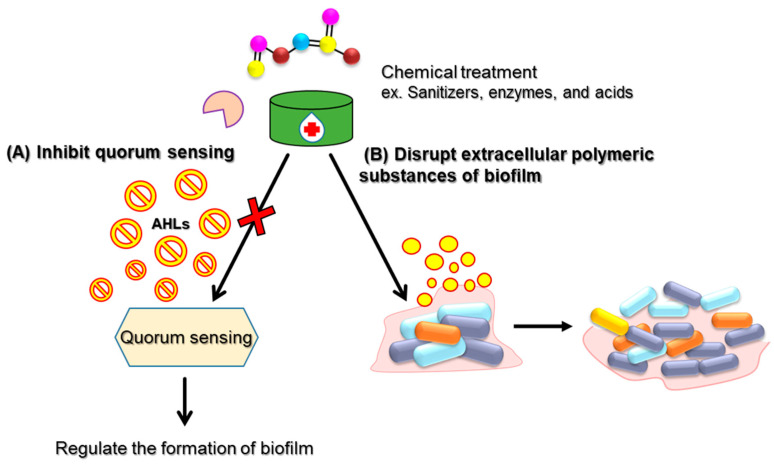
Chemical treatments disrupting quorum sensing systems and biofilm EPS.

As shown in Table 2, chemical treatments play a crucial role in both food processing and biofilm control. In food processing, chemical treatments are employed to sanitize equipment and surfaces, ensure hygiene, and prevent cross-contaminations of food products. Biofilm control strategies typically involve the use of antimicrobials, surfactants, and enzymes to disrupt the biofilm matrix, inhibit bacterial attachment and quorum sensing, and remove existing biofilms (Figure 4). Quorum sensing inhibition disrupts bacterial communication systems that control group behaviors, including biofilm formation and the production of virulence factors. By interfering with these signaling pathways, chemical inhibitors, such as synthetic molecules, plant-derived compounds, or surfactants, prevent bacterial communities from coordinating their growth and activities. These inhibitors, including N-acyl-homoserine lactone (AHL) analogues and cinnamaldehyde, have been shown to interfere with QS in various foodborne pathogens, including *P. aeruginosa*, *L. monocytogenes*, and *E. coli* [10]. Concurrently, the disruption of EPS enhances the effectiveness of antimicrobial agents by allowing better penetration of disinfectants into the biofilm structure. Chemical agents such as proteases, enzymes (e.g., dispersin B), and surfactants such as cetyltrimethylammonium bromide (CTAB) or sodium dodecyl sulfate (SDS) have been demonstrated to degrade EPS components, thus weakening the biofilm’s structural integrity and rendering embedded bacteria more susceptible to antimicrobial treatment [44]. These chemical strategies are particularly beneficial in the food industry, where biofilm formation on food processing surfaces, equipment, and packaging can lead to persistent microbial contamination and spoilage [44]. The combined use of QS inhibitors and EPS disruptors not only helps in controlling microbial growth but also improves the efficiency of cleaning protocols, reducing the risks of contamination and enhancing the overall safety and shelf life of food products. Furthermore, chemical treatments are integrated into food packaging materials and food contact surfaces to prevent bacterial growth and extend shelf life. These chemical treatments not only enhance food safety but also help maintain product quality, freshness, and sensory attributes, preserving the nutritional value and consumer acceptability of food products.

### 3.1. Chemical Agents for Biofilm Control in Food Processing

Quaternary ammonium compounds (QACs), chlorine-based compounds, peracetic acid, and hydrogen peroxide are sanitizers and disinfectants agents that are widely used in food processing and biofilm control due to their antimicrobial properties and efficacy against a broad spectrum of bacteria. QACs, cationic surfactants with antimicrobial properties commonly used as disinfectants and sanitizers in food processing environments, damage bacterial cell membranes, leading to cell leakage and death [54]. Typically, QACs are used in concentrations ranging from 0.1% to 0.5%, depending on the specific formulation and application [54]. The concentration of QACs can be adjusted based on the type of surface being disinfected and the level of microbial contamination. QACs are effective against bacteria, viruses, and fungi, making them versatile tools for biofilm control and surface disinfection [54]. However, long-term exposure to QACs may lead to bacterial resistance and potential adverse effects on human health and the environment, thus requiring proper use and regulatory oversight. Chlorine-based compounds such as sodium hypochlorite (bleach) and chlorine dioxide are strong oxidants used for water disinfection, surface disinfection, and product cleaning in food processing plants [54]. Chlorine effectively destroys microbial cells by oxidizing essential cellular components and disrupting metabolic pathways [45]. Chlorine concentrations typically range from 50 to 200 ppm for routine sanitation, but higher concentrations, such as 500 ppm, may be required for disinfecting heavily contaminated surfaces [45]. Chlorine is highly effective against bacteria, viruses, and protozoa, but may react with organic matter to form potentially harmful disinfection by-products such as chlorinated hydrocarbons, haloacetic acids, and chloramines [45]. Therefore, proper dosing and monitoring are crucial to ensure effective microbial control and minimize health risks. Peracetic acid is a strong oxidizing agent and antimicrobial agent commonly used in food processing for surface disinfection, equipment sanitation, and microbial decontamination [55]. Peracetic acid rapidly penetrates microbial cells, causing irreversible damage to cell membranes and proteins [55]. This disinfectant targets a broad spectrum of microorganisms, such as bacteria, yeasts, molds, and viruses, and is quickly broken down, providing an eco-friendly alternative to chlorine-based disinfectants [55]. Peracetic acid is usually applied at concentrations ranging from 1000 to 5000 mg/L and at temperatures ranging from 40 °C to 60 °C [56]. Hydrogen peroxide is a highly effective oxidative compound that possesses potent antimicrobial properties, demonstrating broad-spectrum activity against a wide range of microorganisms, including bacteria, viruses, fungi, and bacterial spores. Hydrogen peroxide disrupts bacterial cell membranes, generates ROS, and interferes with cellular metabolism, leading to bacterial death [57]. Hydrogen peroxide is commonly used for surface disinfection, water treatment, and bacterial decontamination in food processing facilities [57]. A previous study demonstrated that 0.5% hydrogen peroxide used on spinach, cucumbers, and tomatoes was the most effective at reducing *Salmonella* and *Listeria* spp. [58]. However, its efficacy may be limited by organic matter and catalase-producing microorganisms, requiring higher concentrations or longer contact times to effectively control bacteria. Overall, QACs, chlorine-based compounds, peracetic acid, and hydrogen peroxide are essential chemical agents in food processing and biofilm control, providing effective means of bacterial disinfection and sanitation [54]. However, proper usage, dosing, and regulatory compliance are needed to ensure food safety, quality, and environmental sustainability in the food industry [54].

### 3.2. Acidulants for Biofilm Control in Food Processing

Acidulants, including citric acid, acetic acid, and lactic acid, have gained prominence as effective agents for biofilm control in the food industry [10]. These acidifiers lower the pH of the environment, creating conditions that destabilize biofilms [10]. Citric acid, commonly found in citrus fruits, is widely used in beverages such as fruit juices and soft drinks to provide a tangy flavor and regulate acidity. It also serves as a preservative in canned and pickled foods, such as fruits, vegetables, and jams [10]. Acetic acid, primarily in the form of vinegar, is a key component in the production of pickled vegetables, condiments, and salad dressings, where it serves both as a flavoring agent and a preservative by reducing pH and inhibiting bacterial growth [59]. Lactic acid, produced during the fermentation process by lactic acid bacteria, is crucial in the dairy industry, contributing to the tangy flavor and texture of products such as yogurt, cheese, and sour cream. Additionally, lactic acid plays a role in the fermentation of vegetables, such as in sauerkraut and kimchi, and is used in the meat processing industry for its antimicrobial properties, enhancing food safety and preservation [10]. Collectively, these acidulants are indispensable in ensuring the quality, safety, and stability of a wide range of food products. The acidic environment disrupts the EPSs of biofilms, facilitating their removal from surfaces and equipment [10]. Consequently, the use of acidulants enhances the effectiveness of sanitation procedures, contributing to cleaner and safer food processing environments. By incorporating acidifiers into cleaning routines, food processors can significantly reduce microbial attachment on surfaces, thereby minimizing contamination risks and improving food safety standards [48]. Additionally, acidifiers can target specific problem areas, such as drains and crevices, where biofilms often develop and are more persistent and difficult to remove with standard cleaners [48]. Beyond their antibacterial properties, acidifiers are non-toxic and environmentally friendly [48]. As naturally occurring substances, acidulants like citric acid and lactic acid are biodegradable and safe for use in food processing environments [48].

### 3.3. Enzyme-Based Approaches for Biofilm Control in Food Processing

Enzymes are naturally occurring catalysts, primarily proteins or RNA, that can accelerate chemical reactions without changing the chemical equilibrium between reactants and products or initiate reactions without being consumed or altered [4]. Based on their functional properties, enzymes can be categorized into six classes: ligases or synthetases, hydrolases, lyases, isomerases, oxidoreductases, and transferases. Oxidoreductases and hydrolases have been shown to be the most promising enzymes for degrading biofilms [4]. Anti-biofilm enzymes have the ability to promote cell lysis by targeting the bacterial cells embedded in the biofilm matrix. The main target of anti-biofilm enzymes is the EPS of biofilms, which can destroy the connection between cells, continuously separate cells, degrade the biofilm matrix, and accelerate the destruction of target biofilms [51]. The EPS matrix is mainly composed of proteins, nucleic acids (eDNA and RNA), and polysaccharides. Thus, this implies that a concentration of enzymes such as polysaccharide hydrolase, proteolytic or peptidoglycan hydrolase, and/or deoxyribonuclease (DNase) may degrade the extracellular polysaccharides, proteins, and/or eDNA of a mature bacterial biofilm matrix, respectively [51]. Enzymes that break down polysaccharides often target linkages such as α-1,4-, β-1,4-, or β-1,3-glycosides, which hydrolyze polysaccharides to produce oligosaccharides and/or monosaccharides [4]. DNase inhibits bacterial coagulation, surface adhesion, and horizontal gene transfer by denaturing the eDNA in the biofilm matrix [60]. However, for effective EPS removal, it may be necessary to combine the enzyme with a different substrate or to perform further physical or chemical treatment if the EPS consists of a heterogeneous mixture of macromolecules [51]. Enzymes also play a role in the biochemical breakdown of the matrix molecule, such as in suppressing QS signaling, weakening the surface adhesion of cells, introducing toxins into the cells, encouraging cell lysis, and/or deactivating other enzymes required for microbial growth [4]. However, the use of enzymes for biofilm control does face certain challenges. Environmental factors such as temperature, pH, and enzyme inhibitors can affect both the activity and stability of enzymes [61]. These inhibitors can be naturally occurring compounds, contaminants, or by-products produced during food processing, all of which can negatively affect enzyme function [61]. For instance, factors such as high or low pH levels can alter the enzyme’s active site, reducing its ability to catalyze reactions effectively. In some cases, inhibitors, whether they be metals, specific chemicals, or even certain food components such as polyphenols and tannins, can bind to the enzyme, blocking its active site or interfering with its catalytic mechanism [4]. Ensuring the enzymes remain effective under diverse conditions requires careful formulation and sometimes the use of stabilizing agents [61]. Despite these challenges, ongoing research and technological advancements are focused on improving the stability, efficacy, and cost-effectiveness of enzyme-based solutions.

## 4. Alternative Methods for Biofilm Elimination in Food Industry

Biological treatments for the control and removal of biofilms focus primarily on utilizing natural organisms and their by-products to disrupt and eliminate biofilms in various environments. These treatments, which often involve the use of bacteriophages, natural compounds, and probiotics, provide a sustainable and environmentally friendly alternative to traditional chemical approaches to control biofilms and reduce the risk of antimicrobial resistance in foodborne pathogenic bacteria. A sample of related studies on the use of biological treatment for biofilm control in food systems is shown in Table 3.

### 4.1. Challenges and Potential of Bacteriophages in Food Biofilm Management

**Figure 5:**
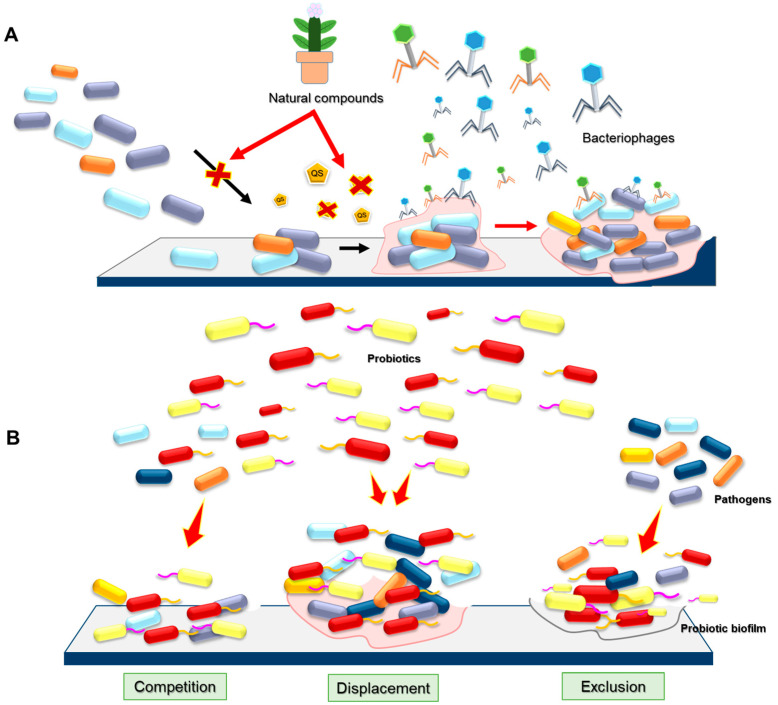
Biological treatments for biofilm control and removal: (**A**) bacteriophages and natural compounds and (**B**) probiotics.

Bacteriophages, viruses that specifically infect bacteria, have emerged as a promising tool for biofilm control in food-related systems [71]. Bacteriophages can target and lyse biofilm-forming bacteria, thereby effectively disrupting and removing the biofilm [72]. Their specificity for bacterial hosts allows for targeted intervention without affecting beneficial microorganisms or the surrounding environment [71]. One of the main advantages of using bacteriophages to control biofilms is their ability to infect and kill bacteria within the biofilm structure [73]. Bacteriophages can penetrate the biofilm matrix and replicate within the bacterial cells, leading to cell lysis and the release of new phage particles that can further infect neighboring bacteria (Figure 5A) [73]. This cycle of infection and lysis can significantly reduce the number of bacteria within the biofilm. Moreover, bacteriophages can be integrated into various stages of food processing to enhance hygiene and safety [74]. They can be applied directly to food contact surfaces, incorporated into cleaning solutions, or even used as a component of packaging materials to prevent biofilm formation and bacterial growth [73]. For example, phage-coated surfaces or phage-embedded films can provide continuous protection against biofilm formation on equipment and packaging, ensuring a safer processing environment [75]. However, despite their potential, bacteriophages face several limitations in biofilm control within food-related systems. One major challenge is the specificity of bacteriophages to their bacterial hosts, which means that a single type of phage might only target one species or strain of bacteria, necessitating the use of phage cocktails to cover a broader range of pathogens [76]. Environmental factors such as temperature, pH, and the presence of other microbial communities can also influence phage activity and stability [77]. Furthermore, there is a risk of bacteria developing resistance to bacteriophages, similar to antibiotic resistance, which could undermine long-term effectiveness [78]. Finally, regulatory hurdles and consumer acceptance of phage treatments in food products and processing environments remain significant barriers to widespread adoption.

### 4.2. Plant-Based and Peptide Solutions for Biofilm Control in Food Safety

Natural compounds, including essential oils, plant extracts, and antimicrobial peptides, have shown efficacy in disrupting biofilm formation and aiding in its removal, improving food safety and hygiene and providing a sustainable and environmentally friendly alternative to synthetic chemicals [79]. Essential oils extracted from plants are rich in bioactive compounds such as terpenes, phenols, flavonoids, and tannins, which have strong antimicrobial properties. These oils can penetrate the biofilm matrix, disrupting the cell membranes of embedded bacteria and inhibiting their growth (Figure 5A). For instance, essential oils from thyme, oregano, and tea trees have demonstrated significant anti-biofilm activity against common foodborne pathogens [80,81]. Extracts from cranberry, garlic, and green tea have been found to inhibit biofilm development by preventing bacterial adhesion and quorum sensing, which are critical processes for biofilm maturation [82,83,84]. Furthermore, the use of plant extracts in food processing environments not only targets biofilms effectively but also aligns with the increasing consumer demand for natural and safe food production methods [79]. Antimicrobial peptides (AMPs), naturally occurring in various organisms, also offer a targeted approach to biofilm control [85]. These short peptides can penetrate biofilms and disrupt bacterial cell walls, leading to cell lysis and biofilm dispersion [86]. AMPs such as nisin, produced by *Lactococcus lactis*, and pediocin, produced by *Pediococcus* species, are already used in food preservation and have demonstrated effectiveness against biofilm-forming bacteria [86]. The incorporation of AMPs into food contact materials or cleaning agents can provide continuous protection against biofilm formation, ensuring a higher level of food safety. However, the efficacy of natural compounds can be affected by environmental conditions such as temperature and pH, which necessitates the development of stable formulations [79]. Regulatory approval for the use of these compounds in food processing environments also requires rigorous safety evaluations. With continued innovation, natural compounds are set to become integral components of biofilm management strategies in the food industry, enhancing food safety and promoting sustainable practices.

### 4.3. Probiotics for Biofilm Control in Food Systems

Probiotics, which are beneficial microorganisms primarily used to promote gut health, are increasing being explored for their potential in controlling biofilm formation in food-related systems [87]. As shown in Figure 5B, probiotics have shown promise in inhibiting biofilm formation and displacing established biofilms by competition, exclusion, and producing bioactive compounds that disrupt biofilm integrity [88]. Competition occurs as probiotics outcompete pathogenic bacteria for essential nutrients, adhesion sites, and space, often producing antimicrobial compounds like bacteriocins and organic acids to suppress biofilm growth [89]. Displacement involves probiotics actively disrupting established biofilms by secreting enzymes (e.g., proteases, DNases) that degrade the biofilm matrix, altering environmental conditions, and replacing pathogenic species with beneficial microbes. Exclusion prevents biofilm formation at the initial stage by blocking pathogen adhesion sites through steric hindrance, modifying surface properties, and producing biosurfactants that reduce bacterial attachment [89].

Certain lactic acid bacteria such as *Lactobacillus plantarum* and *L*. *rhamnosus* have been shown to outcompete foodborne bacteria such as *E*. *coli* and *S*. *aureus* on a variety of surfaces of stainless steel and plastics commonly used in food processing facilities [90,91]. This competition creates a hostile environment for pathogens, reducing their ability to establish strong biofilms [91]. In addition to competitive exclusion, probiotics also produce various antimicrobial substances, including organic acids, hydrogen peroxide, and bacteriocins, which can inhibit the growth of biofilm-forming bacteria and degrade the EPS matrix that protects bacteria within biofilm [92]. For example, bacteriocins produced by *Lactobacillus* spp. have been shown to possess strong antibiofilm activity against common foodborne pathogens [93]. By breaking down the biofilm matrix and killing embedded bacteria, these antimicrobial compounds enhance the effectiveness of cleaning regimens and reduce the risk of contamination [93]. Probiotics provide a safer and environmentally friendly alternative, as they are generally recognized as safe and do not leave harmful residues [89]. The integration of probiotics into cleaning regimens and food contact surfaces presents a promising strategy for maintaining hygiene in food processing environments, ensuring food safety, and protecting public health [89].

## 5. Conclusions

This review underscores the importance of continued innovation and collaboration to overcome the challenges posed by biofilms in the food industry. Biofilms in food processing environments present a persistent threat to food safety and public health due to their ability to protect pathogens from conventional sanitization methods. This issue requires a comprehensive approach that incorporates physical, chemical, and surface modification strategies, approaching the problem from multiple angles. Advances in these areas, such as the use of ultrasound, electric fields, enzymes, and acidulants, have shown promising results in biofilm control and removal. Surface modification techniques further complement these efforts by preventing initial bacterial adhesion and facilitating effective cleaning. While these strategies represent significant progress, further research is needed to optimize their application, scalability, and integration into existing food processing systems. The development of cost-effective, environmentally friendly, and efficient biofilm control technologies will be essential for ensuring food safety and maintaining consumer trust.

## Figures and Tables

**Table 1:** Physical treatment for biofilm control in food industry.

Physical Treatment	Target Strain	Anti-Biofilm Activity	Reference
Thermal	*Staphylococcus aureus*	Superheated steam (SHS) treatment (150 °C, 15 s) can effectively eradicate mature biofilm of *S. aureus* formed on food contact surfaces	[17]
	*Staphylococcus epidermidis*	*S. epidermidis* biofilm cells that formed in liquid egg processing environment were sensitive to hot-water treatment (71 °C).	[18]
Electrical field	Sludge containing mixed-bacteria strains from wastewater treatment plant	Low-frequency and low-voltage range of 8 vpp can stimulate the attachment of the bacteria to solid surfaces and therefore diminish bacterial biofilm-forming ability.	[19]
	*S. epidermidis*	100 μA electric block currents were demonstrated to enable 76% detachment of *S. epidermidis* from stainless steel.	[20]
	*Pseudomonas aeruginosa*, *S. epidermidis*, and *S. aureus*	The biofilm of multiple bacterial species was inhibited by great electricidal effects using low-amperage currents and intermittent strategies.	[21]
Ultrasonic	*S. aureus*	Ultrasonic coda wave interferometry was able to detect the early stage of biofilm formation of on stainless steel.	[22]
	*S. aureus*	The treatment of ultrasonic plus 1% of chlorogenic acid for 60 min exhibited synergistic antibacterial and antibiofilm effects by cause damage to cell morphology and decreasing the exopolysaccharide contents in *S. aureus*.	[23]
	*E. coli* and *L. monocytogenes*	The combination of ultrasound and different organic acids (acetic acid, malic acid, citric acid and lactic acid) can detach bacteria on the surface of lettuce.	[24]
	*Salmonella* spp.	Combination of ultrasound (40 kHz) and acidic electrolyzed water produced a synergistic effect on the reduction of *Salmonella* spp. biofilm formed on stainless steel surfaces.	[25]
Surface modification	*E. coli* and *P. aeruginosa*	Oil-based slippery coatings significantly inhibited bacterial adhesion and reduced biofilm formation on stainless steel surface.	[26]
	*E. coli* and *S. aureus*	Silver and zinc oxide nanoparticle-containing polyester surfaces prevented biofilm formation in both Gram-positive and Gram-negative bacteria	[27]
	*S. enteritidis*	Compared to uncoated surfaces, a reduction in biofilm formation was observed on copper-coated surfaces (3–4 log CFU reduction)	[28]

**Table 2:** Chemical treatment for biofilm control in food industry.

Chemical Treatment	Target Strain	Anti-Biofilm Activity	Reference
Sanitizers and disinfectants	*S. Enteritidis*	Chlorine dioxide showed biofilm removal ability on food contact surfaces (stainless steel, silicone rubber, and plastic) and chicken skin.	[45]
	*L. monocytogenes*	Peracetic acid-based commercial disinfectant showed biofilm formation-inhibitory effect on stainless steel surface at low temperature.	[46]
	*B. cereus.*	Chemical sanitizers containing quaternary ammonium compound (QAC) dramatically reduced the presence of a biofilm on food contact surfaces (glass, polyethylene, polypropylene, and wood).	[47]
Acidulants	*B. subtilis*	2% citric acid showed biofilm removal ability on stainless steel surface.	[48]
	*E. coli* O157:H7	Lactic acid bacteria inhibited growth and surface colonization of *E. coli* O157:H7 at 10 °C.	[49]
	*V. parahaemolyticus* and *P. aeruginosa*	Lactic acid bacteria isolated from Korean fermented vegetable (kimchi) showed biofilm removal ability on seafood model and food contact surfaces (rubber and high-density polyethylene plastic).	[50]
	*V. parahaemolyticus*	Enzymatic cocktail of lipase, cellulase, and proteinase K showed biofilm removal ability by disrupted EPS of bacterial biofilm.	[51]
Enzymes	*E. coli* and *S. aureus*	A novel food packaging material was obtained by immobilizing glucose oxidase in polyvinyl alcohol/chitosan/tea extract electrospun nanofibrous membrane.	[52]
	*Macrococcus caseolyticus*	Enzymatic cocktail of protease, lipase, cellulase, a-amylase, and DNase showed biofilm removal ability in dairy industries by disrupted EPS of bacterial biofilm.	[53]

**Table 3:** Biological treatment for biofilm control in food industry.

Biological Treatment	Target Strain	Anti-Biofilm Activity	References
Bacteriophages	*Cronobacter sakazakii*	Bacteriophages isolated from sewage showed biofilm removal ability in infant formula milk industry by targeting biofilm matrix.	[62]
	*Listeria monocytogenes*	*Listeria*-specific bacteriophage cocktail effectively eradicate matured biofilm formed on food contact materials including polyethylene, polypropylene, and stainless steel.	[63]
	*Aeromonas hydrophila*	Bacteriophages isolated from the sediment of a fish farm inhibited biofilm formation and degraded and killed bacteria in matured biofilms on lettuce.	[64]
Natural compounds	*Staphylococcus aureus* and *Enterococcus faecalis*	Carvacrol isolated from the leaves of wild bergamot showed antibacterial and antibiofilm activities by inhibiting bacterial motility and interfering with bacterial adhesion.	[65]
	*L. monocytogenes*	Cinnamaldehyde, eugenol, resveratrol, and thymoquinone isolated from plants inhibited biofilm formation in beef processing plants by interfering with quorum sensing systems.	[66]
	*Escherichia coli*, *S. aureus*, and *Bacillus pumilus*	Thymol and eugenol can be used to prepare novel active food packaging for the dairy industry to prevent biofilm formation.	[67]
Probiotics	*S. aureus*, *L. monocytogenes*, and *S.* Typhimurium	*Lactobacillus plantarum* isolated from Korean fermented kimchi showed antibiofilm formation by inhibiting bacterial adhesion to surface.	[68]
	*S.* Enteritidis	Bacteriocins produced from *Bacillus subtilis* and *B. amyloliquefaciens* inhibited biofilm formation in poultry products by interfering with the quorum sensing system.	[69]
	*S. aureus*	Probiotic biofilms of *Lactiplantibacillus plantarum* and *Lacticaseibacillus rhamnosus* prevented biofilm formation of bacteria in milk and yogurt during processing and storage conditions.	[70]

## Data Availability

No new data were created or analyzed in this study. Data sharing is not applicable to this article.
